# Preliminary Analysis of the Impact of Malaria Vaccine Introduction on Severe Malaria Incidence in Children Under 5 Years in the Health Districts of Edea, Japoma, and Mbanga (2020–2024)

**DOI:** 10.1002/hsr2.73475

**Published:** 2026-11-04

**Authors:** Alex Stéphane Ndjip Ndjock, Michaela Josee Meli, Nina Ngouenko Nkakoua, Steve Roland Souga, Prince Théophile Djomou, Marie‐Michelle Ekedy‐Lea, Esther Laura Nyame Dipepa Nwall

**Affiliations:** ^1^ Department of Public Health Edea Health District Littoral Region Cameroon; ^2^ Faculty of Medicine and Pharmaceutical Sciences University of Dschang Dschang Cameroon; ^3^ Regional Technical Group for Malaria Control Littoral Regional Public Health Delegation Douala Cameroon; ^4^ Cameroon Society of Epidemiology (Case) Yaoundé Cameroon; ^5^ Expanded Program on Immunization Ministry of Health Yaoundé Cameroon; ^6^ Faculty of Medicine and Pharmaceutical Sciences University of Douala Douala Cameroon

**Keywords:** Cameroon, Children under five (U5), Littoral Region, Malaria vaccine (MV), Pediatric malaria, Severe malaria, Vaccination coverage

## Abstract

**Background and Aims:**

The RTS,S/AS01 malaria vaccine (MV) was introduced in Cameroon on 22 January 2024 in 42 of 200 health districts, including three in the Littoral Region (Edea, Japoma and Mbanga). This study describes early trends in severe malaria among children under 5 years of age in these three districts of the Littoral Region and explores their relation to district‐level MV coverage.

**Methods:**

Retrospective ecological time‐trend study of routine DHIS 2 data, 2020–2024. Annual confirmed severe and uncomplicated malaria cases were described by district; 2024 was compared with 2023 and with the 2020–2023 mean. MV1–MV3 coverage and dropout were computed for 2024. MV3 coverage and the percentage change in severe cases (n = 3 districts) were explored with Pearson's correlation (exact p‐value and 95% confidence interval) and Spearman's rank correlation.

**Results:**

Severe cases rose by 11.2% in 2024 (6,106 to 6,792) after declines of 4.7% in 2022% and 7.0% in 2023; the 2024 total was 10.3% above the 2020–2023 mean, and the rise was smaller than that between 2020 and 2021 (+ 35.8%). Increases were 14.4% in Edea, 11.5% in Japoma and 4.2% in Mbanga. MV3 coverage was 37.5%, 28.8% and 32.0%, respectively; 44.6% of children who received MV1 had not received MV3 (range 33.8%–59.3%). MV3 coverage was not informatively associated with the change in severe cases (Pearson r = 0.41, 95% CI − 0.92 to 0.97, *p* = 0.73; Spearman ρ = 0.50, exact p = 1.00); with three districts the analysis has virtually no power.

**Conclusion:**

No change in severe malaria attributable to the vaccine was detectable in its first year, as expected given recent introduction and low, incomplete coverage. The 2024 increase lies within earlier year‐to‐year variation and cannot be attributed to, or against, the vaccine. Dropout between the first and third doses is substantial. Impact evaluation requires monthly time‐series analyses with comparison districts, population denominators and longer follow‐up.

## 1 Introduction

Malaria remains one of the world's leading public health problems, with a particularly high burden in sub‐Saharan Africa, where most cases and deaths occur. Children under 5 years of age are the most vulnerable because their immunity is still immature, which exposes them to severe disease and death [1]. Despite progress over recent decades, reductions in malaria incidence and mortality have slowed in several endemic countries, signaling a plateau in gains and the limits of current control strategies [2].

In Cameroon, malaria is hyperendemic and transmission is intense and perennial in the forested and coastal south, notably in the Littoral Region, whereas it is markedly seasonal in the Sudano‐Sahelian North and Far North regions [3, 4]. Control relies on long‐lasting insecticidal nets, rapid diagnostic testing, artemisinin‐based combination therapy and intermittent preventive treatment in pregnancy. In addition, Cameroon adopted seasonal malaria chemoprevention (SMC) in 2016 and has delivered it every year since, to children under 5 years in the North and Far North regions, with campaigns between July and October [4]. SMC is therefore not part of the package in the Littoral Region. Despite these interventions, malaria, particularly its severe forms, continues to place a heavy burden on the health system.

In this context, malaria vaccination is a major advance. The RTS,S/AS01 vaccine showed moderate but significant efficacy against clinical and severe malaria in phase 3 trials in African children, and the R21/Matrix‐M vaccine has since shown higher efficacy in randomized trials, although results depend on the transmission setting. On this basis, the World Health Organization (WHO) recommended RTS,S/AS01 in 2021 for children living in areas of moderate to high transmission [5, 6, 7, 8].

Cameroon introduced RTS,S/AS01 into its routine immunization program on 22 January 2024, in the 42 districts (of 200) judged most at risk, using a four‐dose schedule at 6, 7, 9 and 24 months of age [9]. The first 30 days of rollout showed good acceptance, but also low coverage and incomplete data reporting in some regions [9]. The impact of the vaccine will depend on the performance of the routine immunization system into which it is integrated [10], and this system faces logistical and informational constraints in Cameroon. Logistical constraints include access to vaccination services, vaccine availability, waiting times and perceived quality of services; informational constraints include limited communication between caregivers and health workers. National coverage of the third dose of diphtheria‐tetanus‐pertussis vaccine reportedly fell from 89% in 2013% to 67% in 2019, and a survey of 438 caregivers in urban Buea found that 25.1% of children were under‐immunized; under‐immunization was associated with caregivers not having received informal advice on vaccination and not perceiving an improvement in the quality of vaccine services, and only 59.4% of caregivers had discussed vaccination concerns with health workers [11]. The malaria vaccine schedule adds contacts at 6 and 7 months of age, which may amplify these constraints and make completion of the series more difficult. In sub‐Saharan Africa more broadly, coverage is influenced by structural, organizational and socio‐economic factors, including service accessibility, quality of care and population adherence [12, 13, 14, 15].

Real‐world data on the impact of the malaria vaccine are scarce in Africa and almost absent in Cameroon [9, 10]. This study therefore describes trends in severe malaria among children under 5 years of age in the health districts of Edea, Japoma and Mbanga (Littoral Region) over 2020–2024, and explores, at district level, their relationship with vaccine coverage in the first year of rollout.

## 2 Methods

### 2.1 Study Design

This was a retrospective, ecological time‐trend study using routine health surveillance data. The unit of analysis was the district‐year. Changes in malaria morbidity were examined before (2020–2023) and after (2024) the introduction of the malaria vaccine. The study is reported in line with the STROBE and RECORD recommendations [16, 17].

### 2.2 Study Setting

The study was conducted in three health districts of the Littoral Region of Cameroon: Edea, Japoma and Mbanga. These districts were purposively selected because they were among the 42 health districts nationally enrolled in the first phase of the malaria vaccine rollout in 2024.

The Littoral Region is characterized by a coastal and forested ecology with high, perennial malaria transmission. Malaria is hyperendemic and *Plasmodium falciparum* is the predominant species. The population is predominantly urban and semi‐urban, with mixed economic activities including commerce, industry and agriculture. Malaria control interventions in these districts before vaccine introduction included insecticide‐treated net (ITN) distribution, intermittent preventive treatment in pregnancy (IPTp), and prompt diagnosis and treatment with artemisinin‐based combination therapy (ACT) at community and facility levels. SMC is implemented in Cameroon only in the North and Far North regions [4]; it was not delivered in the study districts and therefore could not be included in the analysis. The malaria vaccine was delivered through routine immunization services on a four‐dose schedule at 6, 7, 9 and 24 months of age, from 22 January 2024 [9].

### 2.3 Study Population

The study population comprised all children under 5 years of age (0–59 months) residing in the three health districts who presented to any public or private health facility and received a confirmed diagnosis of malaria (uncomplicated or severe) during the study period. For vaccination coverage, the target population comprised all children eligible for the malaria vaccine according to the national immunization schedule, using the district denominators recorded in DHIS 2.

### 2.4 Data Sources and Data Quality

Data were extracted from the District Health Information Software 2 (DHIS 2), the national routine health information system of Cameroon, for 1 January 2020 to 31 December 2024: (i) monthly confirmed malaria cases in children under 5 years, disaggregated into severe malaria (confirmed malaria with one or more signs of severity according to the national case definition) and uncomplicated malaria (confirmed malaria without signs of severity); and (ii) monthly vaccination data for 2024, comprising the number of children who received each dose of the malaria vaccine (MV1, MV2, MV3) and the corresponding target population denominators. Operational definitions are given in Table 1.

**Table 1:** Operational definitions.

Term	Definition
Confirmed malaria case	A case with a positive malaria diagnostic test (microscopy or rapid diagnostic test [RDT]) in a child presenting with fever or a history of fever
Severe malaria	Confirmed malaria with one or more clinical or laboratory signs of severity (e.g., impaired consciousness, respiratory distress, severe anemia [hemoglobin < 5 g/dL], jaundice, hypoglycemic, repeated convulsions, prostration, or inability to drink)
Uncomplicated malaria	Confirmed malaria in the absence of any signs of severity
MV1/MV2/MV3	First, second and third doses of the malaria vaccine, administered according to the national schedule (four doses at 6, 7, 9 and 24 months of age; the fourth dose was not assessed)
MV coverage	(Number of children vaccinated with the dose/target population of the district) × 100
Vaccination dropout	Proportion of children who received MV1 but not MV3: [(MV1 − MV3)/MV1] × 100, calculated from absolute numbers
Percentage change	[(Cases in year Y − cases in year Y − 1)/cases in year Y − 1] × 100
Pre‐vaccination baseline	Mean annual number of cases over 2020–2023

Before entry into DHIS 2, monthly facility reports were validated at district level through the national surveillance routine. The checks comprised: (i) completeness and timeliness, confirming that a monthly report had been received from each public and private reporting facility; (ii) internal consistency between related data elements (e.g., severe cases cannot exceed confirmed cases, confirmed cases cannot exceed the number of children tested, and the number of children receiving MV2 or MV3 cannot exceed the number receiving MV1); (iii) detection of outliers by comparison with the facility's preceding months and with the same month in previous years, with queries returned to the facility for verification against source registers and correction; and (iv) review and validation of the consolidated district report at the monthly district data review meeting, followed by entry into DHIS 2, whose input validation flags out‐of‐range values. These checks follow the dimensions of the WHO data quality framework (completeness, timeliness and internal consistency) [18]. For this study, we additionally verified that annual aggregates equalled the sum of district values and that dose‐specific vaccination counts decreased from MV1 to MV3 in every district.

### 2.5 Variables

#### 2.5.1 Outcomes

The annual number of confirmed severe malaria cases and of confirmed uncomplicated malaria cases in children under 5 years, by district and calendar year (2020–2024). Because the analysis used reported case counts without population denominators, “incidence” in this paper refers to the number of reported cases and not to a population‐based rate.

#### 2.5.2 Exposure

Malaria vaccine coverage, defined as the percentage of the target population who received each dose (MV1, MV2, MV3): (number of children vaccinated with the dose/target population of the district) × 100.

#### 2.5.3 Derived Variables

Year‐on‐year percentage change in cases; percentage change in severe cases in 2024 relative to 2023; percentage change in 2024 relative to the 2020–2023 mean (pre‐vaccination baseline); and vaccination dropout between MV1 and MV3, [(MV1 − MV3)/MV1] × 100, calculated from the absolute numbers of children vaccinated.

### 2.6 Statistical Analysis

Data were exported from DHIS 2 to Microsoft Excel (version 16.0) for cleaning and organization. Analyses were performed in R (version 4.2.1). Three steps were taken.

- *Descriptive analysis*. Annual case counts for severe and uncomplicated malaria were tabulated by district and for the three districts combined.
- *Trend analysis*. Year‐on‐year percentage change was calculated as [(cases in year Y − cases in year Y − 1)/cases in year Y − 1] × 100, for severe and uncomplicated cases. The 2024 figures were compared with the 2020–2023 mean and placed in the context of the range of annual changes observed in 2021–2023.
- *Exploratory correlation analysis*. Pearson's correlation coefficient (r) was calculated between MV3 coverage in 2024 (independent variable) and the percentage change in severe cases from 2023 to 2024 (dependent variable) across the three districts. With n = 3 there is one degree of freedom, so the two‐sided p‐value was obtained from the exact t distribution with n − 2 degrees of freedom, and the 95% confidence interval was obtained by inverting the exact sampling distribution of r [19], because Fisher's z method is not defined for n = 3. Spearman's rank correlation (exact p‐value) was added as a sensitivity analysis. Given the sample size, these estimates were interpreted as exploratory and hypothesis‐generating; no thresholds were applied to judge the strength of association, and no causal inference was drawn. Multivariable adjustment was not possible.

Although monthly case counts were extracted, the analysis was conducted on annual totals: vaccination data were available only as annual 2024 figures, and the first post‐introduction year, during which coverage accrued progressively, provides only 12 monthly observations. Interrupted time‐series analysis of monthly data is discussed below as the appropriate next step. All tests were two‐sided with a significance level of 0.05.

## 3 Results

### 3.1 Trends in Severe and Uncomplicated Malaria, 2020–2024

In the three districts combined, confirmed severe malaria cases in children under 5 years rose from 5,071 in 2020 to a peak of 6,885 in 2021 (+ 35.8%), declined to 6,563 in 2022 (− 4.7%) and 6,106 in 2023 (− 7.0%), then increased to 6,792 in 2024 (+ 11.2%) (Table 3, Figure 1A). The 2024 count was 10.3% above the 2020–2023 mean (6,156) and 1.4% below the 2021 peak. Edea and Japoma showed an overall upward trend for severe cases between 2020 and 2024, whereas Mbanga recorded consistently lower figures (Table 2). In 2024, Japoma had the highest number of severe cases (2,778, against 2,773 in Edea). Compared with 2023, severe cases rose by 14.4% in Edea, 11.5% in Japoma and 4.2% in Mbanga; compared with the 2020–2023 mean, the changes were +15.5%, +11.3% and −1.5%, respectively (Table 6). Before vaccine introduction, annual changes ranged from −7.0% to +35.8% in the combined data and from −17.8% to +53.9% at district level.

**Figure 1:**
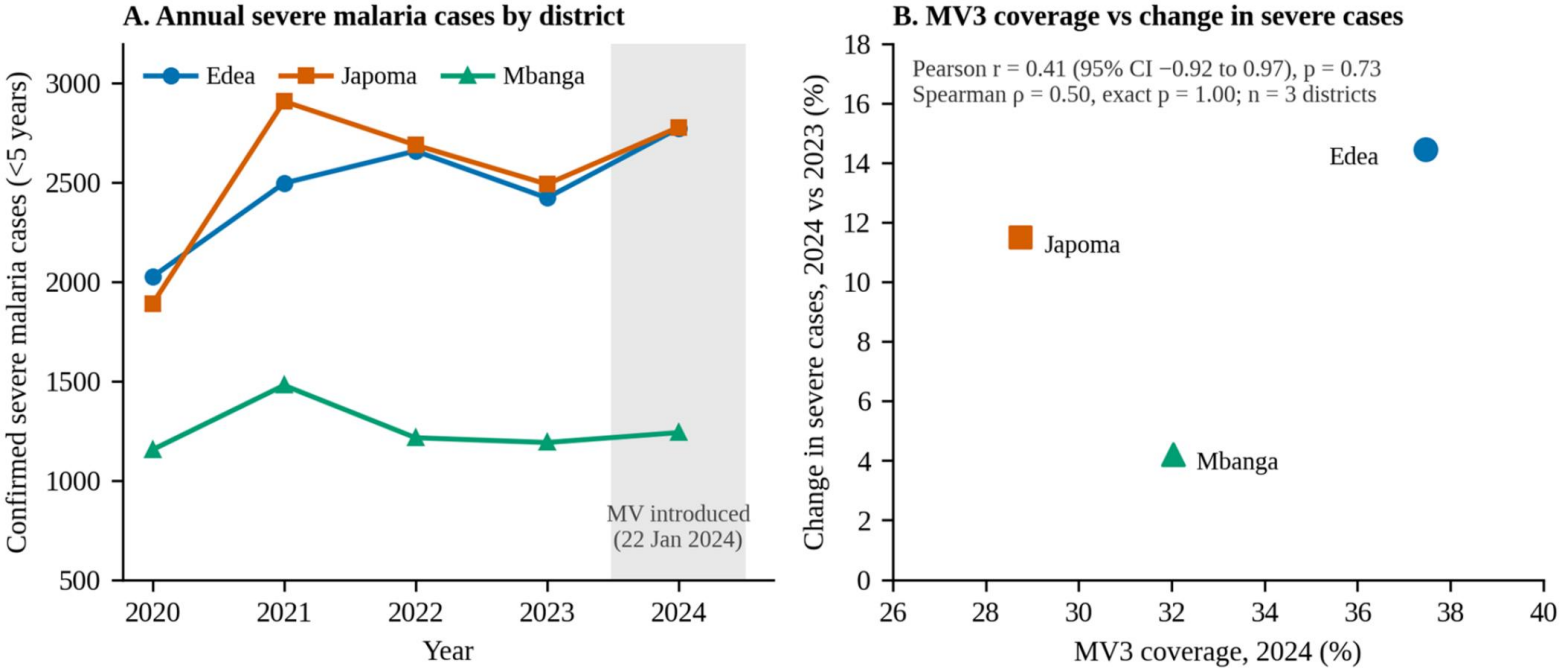
(A) Annual confirmed severe malaria cases in children under 5 years, by district, 2020–2024; the shaded band marks 2024, the year of malaria vaccine (MV) introduction (22 January 2024). (B) MV3 coverage in 2024 versus percentage change in severe cases between 2023 and 2024 in the three districts; the statistics are exploratory and, with n = 3, uninformative.

**Table 2:** Annual evolution (2020–2024) of confirmed severe and uncomplicated malaria cases in children under 5 years, by district.

	Confirmed severe malaria cases (< 5 years)					Confirmed uncomplicated malaria cases (< 5 years)				
**District**	**2020**	**2021**	**2022**	**2023**	**2024**	**2020**	**2021**	**2022**	**2023**	**2024**
Edea	2,026	2,497	2,659	2,423	2,773	2,273	2,478	2,895	2,594	2,789
Japoma	1,890	2,909	2,689	2,492	2,778	2,155	2,548	2,886	2,447	2,385
Mbanga	1,155	1,479	1,215	1,191	1,241	1,480	1,370	1,793	2,205	828

Uncomplicated cases in the three districts combined peaked in 2022 (7,574) and fell to 6,002 in 2024 (− 17.2% *vs.* 2023) (Table 3). This fall was concentrated in Mbanga, where uncomplicated cases dropped from 2,205 in 2023 to 828 in 2024 (− 62.4%), whereas they changed little in Edea (+7.5%) and Japoma (−2.5%). In 2024, Edea had the highest number of uncomplicated cases (2,789) (Table 2).

**Table 3:** Annual aggregation (2020–2024) of malaria cases in children under 5 years in the three districts combined (Edea, Japoma, Mbanga).

Year	Severe cases	% change vs previous year	Uncomplicated cases	% change vs previous year
2020	5,071	—	5,908	—
2021	6,885	+35.8	6,396	+8.3
2022	6,563	−4.7	7,574	+18.4
2023	6,106	−7.0	7,246	−4.3
2024	6,792	+11.2	6,002	−17.2
Mean 2020–2023	6,156		6,781	
2024 vs mean 2020–2023		+10.3		−11.5

### 3.2 Vaccine Coverage and Dropout, 2024

Coverage fell markedly between the first and third doses in all districts (Table 4). Japoma had the highest MV1 coverage (71.89%) but the lowest MV3 coverage (28.75%); Edea had the highest coverage for MV2 (48.08%) and MV3 (37.46%); and Mbanga had the lowest coverage for MV1 (51.29%) and MV2 (39.19%). In absolute numbers (Table 5), 8,310 children received MV1 and 4,606 received MV3 across the three districts. Dropout between MV1 and MV3 was 33.8% in Edea (1,255 children), 59.3% in Japoma (1,967) and 37.7% in Mbanga (482), and 44.6% overall (3,704 children).

**Table 4:** Coverage rate (%) by dose of malaria vaccine (MV) in each district, 2024.

District	MV1 coverage	MV2 coverage	MV3 coverage
Edea	64.54	48.08	37.46
Japoma	71.89	44.41	28.75
Mbanga	51.29	39.19	32.03

**Table 5:** Number of children vaccinated against malaria by dose and district, and dropout between MV1 and MV3, 2024.

District	MV1 (n)	MV2 (n)	MV3 (n)	MV1 − MV3 (n)	Dropout MV1 → MV3 (%)
Edea	3,712	2,846	2,457	1,255	33.8
Japoma	3,318	2,067	1,351	1,967	59.3
Mbanga	1,280	985	798	482	37.7
Total	8,310	5,898	4,606	3,704	44.6

*Note:* Dropout was calculated from absolute numbers of children, as [(MV1 − MV3)/MV1] × 100.

### 3.3 Exploratory Analysis of MV3 Coverage and Change in Severe Cases

MV3 coverage was highest in Edea (37.46%), which also had the largest increase in severe cases (+14.4%); Japoma had the lowest MV3 coverage (28.75%) and an increase of +11.5%; Mbanga had intermediate coverage (32.03%) and the smallest increase (+4.2%) (Table 6, Figure 1B). The correlation between MV3 coverage and the percentage change in severe cases was r = 0.41 (95% CI − 0.92 to 0.97; exact *p* = 0.73); Spearman's ρ was 0.50 (exact p = 1.00). The point estimate is positive, that is, not in the direction that would be expected if higher completion were associated with smaller increases, but the confidence interval spans almost the entire possible range, from strongly negative to strongly positive correlation. With three observations, these results are uninformative about any association.

**Table 6:** Severe malaria cases, percentage change and MV3 coverage by district, with exploratory correlation.

District	Severe cases 2023	Severe cases 2024	% change 2024 vs 2023	% change 2024 vs mean 2020–2023	MV3 coverage 2024 (%)
Edea	2423	2773	+14.4	+15.5	37.46
Japoma	2492	2778	+11.5	+11.3	28.75
Mbanga	1191	1241	+4.2	−1.5	32.03
Three districts	6106	6792	+11.2	+10.3	—

*Note:* Exploratory correlation between MV3 coverage and % change in severe cases 2024 vs 2023 (n = 3 districts): Pearson r = 0.41, exact 95% CI − 0.92 to 0.97, exact two‐sided *p* = 0.73; Spearman ρ = 0.50, exact two‐sided p = 1.00. With one degree of freedom, these estimates are uninformative and are not evidence for or against an association.

## 4 Discussion

This preliminary analysis describes severe malaria trends in children under 5 years in three Littoral districts during the first year of malaria vaccine rollout. Severe cases rose by 11.2% in 2024 relative to 2023 but remained 1.4% below the 2021 peak. Vaccine coverage was modest (MV3: 28.8%–37.5%), and 44.6% of children who started the schedule had not received MV3. Across the three districts, MV3 coverage was not informatively related to the change in severe cases. These data therefore cannot be used to judge the impact of the vaccine, whether favorable or unfavorable.

Several features of the rollout make a detectable population‐level effect on severe malaria in 2024 unlikely. Vaccination began on 22 January 2024 [9]; under the 6‐, 7‐ and 9‐month schedule, a child could receive MV3 about 3 months after MV1 at the earliest, so protection from the full primary series could only accrue during the year, and the fourth dose at 24 months was not yet due for any child. The vaccine targets children from 6 months of age, whereas the outcome covers all children aged 0–59 months, and no district had reached 40% MV3 coverage. Efficacy against severe malaria in trials is moderate [6, 7, 8], effectiveness is typically assessed after completion of the primary series and with at least a year of follow‐up [20], and modeling suggests that population‐level impact depends strongly on coverage and completeness of vaccination [21]. Early country experiences, such as that of Sierra Leone, have focused on implementation rather than impact [22], and benefits are expected to become detectable only with sufficient observation time and high, homogeneous coverage [10, 23]. The absence of an early signal is therefore what would be expected.

The 2024 rise should also be read against the year‐to‐year variability seen before vaccine introduction: annual changes during 2021–2023 ranged from −7.0% to +35.8% in the combined data and from −17.8% to +53.9% at district level, and the district increases in 2024 (+ 4.2% to +14.4%) fall within this range. The persistently high burden is consistent with the slowdown of progress across sub‐Saharan Africa [2] and with cohort data from Ghana and Kenya showing high, sustained incidence in children under five before vaccine rollout [24]. Structural factors such as limited coverage of vector control and inequalities in access to early care, which condition progression to severe forms, may contribute [3, 10]. Rainfall and temperature, the timing of net distribution, health‐seeking behavior and health system disruptions, none of which we could measure, may also have modulated transmission and case detection in 2024.

The 62.4% fall in uncomplicated cases in Mbanga in 2024 (2,205 to 828), with stable numbers of severe cases, is atypical and unlikely to reflect a true epidemiological change, particularly with MV3 coverage of 32% in that district. Abrupt shifts of this kind in routine data more often reflect changes in reporting completeness, testing or diagnostic practices, or disruptions to surveillance [9, 25]. Because Mbanga also contributes the smallest rise in severe cases, and the district‐level comparison rests on only three observations, an artifact in Mbanga's 2024 data could by itself alter the apparent pattern, and an audit of its 2024 reports against source registers is warranted. The same observation tempers the interpretation of the overall decline in uncomplicated cases (−17.2%): it was concentrated in Mbanga and we do not interpret it as evidence of a change in disease progression.

Vaccination dropout was high in all three districts. Japoma recorded the highest MV1 coverage (71.9%) but the lowest MV3 coverage (28.8%), a pattern that points to loss to follow‐up rather than lack of initial demand. Attrition between first and last doses is widely documented in routine immunization in sub‐Saharan Africa [12, 13, 14, 15]. Reported determinants include geographical accessibility, indirect costs, quality of care and caregiver perceptions [14, 26, 27]; comparable logistical and informational constraints have been described in Cameroon [11], and early malaria vaccine rollout was marked by low coverage and incomplete reporting [9]. A schedule requiring contacts at 6, 7, 9 and 24 months may amplify these constraints, because program features such as the number of doses and visits influence delivery performance [28]. The fourth dose, important for sustained protection, was outside the scope of this analysis.

We examined whether districts with higher MV3 coverage experienced smaller increases in severe cases. With three districts, this comparison cannot support inference: Pearson's r was 0.41 (95% CI − 0.92 to 0.97; exact *p* = 0.73) and Spearman's ρ was 0.50 (exact p = 1.00). The interval is compatible with strongly negative as well as strongly positive correlation. The point estimate is positive: Edea had the highest MV3 coverage and the largest increase, and Mbanga an intermediate coverage and the smallest increase. Neither pattern should be over‐interpreted, since ecological correlations are vulnerable to the ecological fallacy and to confounding by district‐level characteristics, and percentage changes in districts of different size are sensitive to small absolute differences [29]. Our findings therefore neither support nor refute a dose–response relationship between completion of the vaccination schedule and severe malaria.

Evaluating impact will require designs with greater power and better control of secular trends and confounding. These include controlled interrupted time‐series analysis of monthly district data [30], with adjustment for seasonality and comparison districts that have not introduced the vaccine (only 42 of 200 districts were enrolled in the first phase [9]); inclusion of all vaccinating districts, to allow a meaningful dose–response analysis; population‐based rates rather than counts; individual‐level designs linking vaccination status to case records; and longer follow‐up that covers the fourth dose. Strengthening surveillance and data quality is a prerequisite, since the quality of routine data determines the validity of epidemiological analyses in resource‐limited settings [18, 25].

### 4.1 Strengths and Limitations

This study provides early real‐world data from Cameroon on severe malaria and vaccine delivery, covers all three vaccinating districts of the Littoral Region, uses four pre‐introduction years as a comparator, and reports statistical uncertainty transparently. Its limitations are as follows.

- *Very small number of units*. With three districts, correlation estimates are extremely imprecise, the assumptions of Pearson's correlation cannot be verified, and the results are exploratory only.
- *Ecological design and confounding*. The study could not control for climatic, ecological, programmatic or health‐seeking factors (e.g., rainfall, vector control coverage, testing practices, health system disruptions) that may have influenced transmission and case detection. The 2024 changes cannot be attributed to the vaccine.
- *Short follow‐up and annual aggregation*. Only one post‐introduction year was available, during which coverage was still accruing and the fourth dose was not yet due; annual totals hide seasonality, and interrupted time‐series analysis of monthly data was not performed.
- *Counts rather than rates*. No population denominators were used for cases, so changes in catchment population or in the number of reporting facilities may have affected the counts; percentage changes are sensitive to baseline in small districts.
- *Aggregated data*. The absence of individual‐level data precluded estimation of vaccine effectiveness, comparison of vaccinated and unvaccinated children, and adjustment for individual confounders (age, sex, nutritional status, ITN use). The age distribution of cases relative to vaccine eligibility is unknown.
- *Data quality*. As with all routine information systems, under‐reporting, misclassification, and variation in diagnostic practices across facilities and over time cannot be excluded, as illustrated by the atypical 2024 data from Mbanga. No formal data quality audit was conducted, and coverage denominators are subject to uncertainty.
- *Generalizability*. Three purposively selected districts of a single region limit generalization to other settings.

Despite these limitations, routine data offer valuable real‐world evidence on the early performance of the vaccine program and highlight areas requiring further investigation.

## 5 Conclusion and Recommendations

This preliminary analysis of routine surveillance data from three districts of the Littoral Region found that severe malaria cases in children under 5 years increased by 11.2% in 2024, the year of malaria vaccine introduction, an increase within the range of year‐to‐year variation observed in 2020–2023. Coverage of the full primary series was low (28.8%–37.5%), and dropout between the first and third doses was high (33.8%–59.3%). Across the three districts, MV3 coverage showed no informative relationship with the change in severe cases (r = 0.41, 95% CI − 0.92 to 0.97; *p* = 0.73), and the design cannot attribute the 2024 increase, or any change, to the vaccine. These findings should be seen as a baseline for evaluation rather than as evidence of impact.

The results have the following implications for policy and practice:

- **Improve completion of the schedule.** The high dropout observed, particularly in Japoma, warrants action irrespective of the correlation analysis: defaulter tracing, reminder and recall systems [13], outreach and community health worker engagement [27], and targeted communication with caregivers on the visits required.
- **Strengthen data quality.** Mbanga's 2024 reports should be audited, and routine data quality reviews and triangulation with other sources (health facility assessments, community surveys) should be maintained.
- **Evaluate impact with appropriate designs.** Monthly controlled interrupted time‐series analyses with comparison districts, population denominators, at least 24 months of follow‐up covering the fourth dose, and individual‐level linkage of vaccination status to case records.
- **Continue integrated malaria control.** The vaccine is a complementary tool, not a replacement for existing interventions. Sustained investment in ITNs, IPTp, and prompt case management remains critical, as does SMC in the regions where it is indicated.

The contribution of the malaria vaccine to reducing severe malaria will depend on the strength of the health system that delivers it and of the data systems that monitor it. As Cameroon and other endemic countries scale up malaria vaccination, rigorous evaluation, using both routine surveillance and dedicated studies, will be essential to guide evidence‐based policy.

## Author Contributions

**Alex Stéphane Ndjip Ndjock:** conceptualization, investigation, writing – original draft, methodology, validation, visualization, writing – review and editing, software, formal analysis, data curation, supervision, resources, project administration. **Michaela Josee Meli:** writing – original draft, writing – review and editing, formal analysis, visualization, data curation, methodology. **Nina Ngouenko Nkakoua:** formal analysis, writing – review and editing, data curation, supervision. **Steve Roland Souga:** supervision, writing – review and editing, project administration. **Prince Théophile Djomou:** writing – review and editing, supervision. **Marie‐Michelle Ekedy‐lea:** visualization, writing – review and editing, validation, formal analysis. **Esther Laura Nyame Dipepa Nwall:** supervision, project administration, writing – review and editing.

## Funding

The authors have nothing to report.

## Ethics Statement

This study used anonymized aggregated routine surveillance data extracted from the national DHIS 2 platform. No patient‐identifiable information was accessed or analyzed. As the study involved no direct patient contact, intervention, or collection of biological samples, it was exempt from formal ethical review in accordance with Cameroonian national regulations governing the use of routine de‐identified health data for operational research. The research was conducted in compliance with the principles of the Declaration of Helsinki and received administrative authorization from the Littoral Regional Health Delegation and the respective district health authorities.

## Consent

The authors have nothing to report. The manuscript does not contain any individual person's data in any form (including individual details, images, or videos).

## Conflicts of Interest

The authors declare no conflicts of interest.

## Transparency Statement

The lead author and manuscript's guarantor, Alex Stéphane Ndjip Ndjock, affirms that this manuscript is an honest, accurate, and transparent account of the study being reported; that no important aspects of the study have been omitted; and that any discrepancies from the study as planned (and, if relevant, registered) have been explained.

## Data Availability

The data that support the findings of this study are available from the corresponding author upon reasonable request.
